# Registered nurses’ experiences of patient violence on acute care psychiatric inpatient units: an interpretive descriptive study

**DOI:** 10.1186/s12912-015-0079-5

**Published:** 2015-05-17

**Authors:** Kelly N. Stevenson, Susan M. Jack, Linda O’Mara, Jeannette LeGris

**Affiliations:** St. Joseph’s Healthcare Hamilton, Hamilton, ON Canada; School of Nursing, McMaster University, Hamilton, ON Canada

**Keywords:** Patient violence, Aggression, Inpatient psychiatry, Registered Nurse, Interpretive description

## Abstract

**Background:**

Nurses working in acute care psychiatry settings experience high rates of patient violence which influences outcomes for nurses and the organization. This qualitative study explored psychiatric nurses’ experiences of patient violence in acute care inpatient psychiatric settings.

**Methods:**

An interpretive descriptive design guided this study that included 17 semi-structured interviews with a purposeful sample of 12 Canadian registered nurses who self-reported experiencing patient violence within acute care inpatient psychiatry. Thematic analysis and constant comparison techniques were used for analysis. A problem, needs and practice analysis was also used to structure overall data interpretation.

**Results:**

Thirty three unique exposures to patient violence among the sample of nurses were analysed. Nurses reported experiencing physical, emotional and verbal violence. For many, patient violence was considered “part of the job.” Nurses often struggled with role conflict between one’s duty to care and one’s duty to self when providing care following a critical incident involving violence. Issues of power, control and stigma also influenced nurse participant perceptions and their responses to patient violence. Nurses used a variety of strategies to maintain their personal safety and to prevent, and manage patient violence. Nurses endorsed the need for improved education, debriefing following an incident, and a supportive work environment to further prevent patient violence. Present findings have implications for reducing the barriers to reporting violent experiences and the creation of best practice guidelines to reduce patient violence in the workplace.

**Conclusions:**

Understanding the perspectives and experiences of nurses in acute inpatient psychiatry leads to greater understanding of the phenomenon of patient violence and may inform the development of interventions to prevent and to respond to patient violence, as well as support nurses working within the acute care setting.

## Background

Registered nurses (RNs), compared to other healthcare providers are at a higher risk of experiencing violence in the workplace [1, 2] that is initiated by patients and families [3, 4]. Between 25 and 80 % of nurses working in acute care hospitals have reported experiencing patient violence in one form or another [2, 5, 6] with existing literature hypothesizing that these events are vastly underreported [3, 7, 8].

In Canada, it has been reported that almost one third (29 %) of nurses working in direct care hospitals or long term care facilities reported a physical assault by a patient in the last 12 months, and 44 % reported having experienced emotional abuse [9]. Specifically within the psychiatric nursing population, Hesketh and colleagues [3] reported that 55 % of Canadian psychiatric nurses were victims of verbal or emotional abuse, 19.5 % experienced sexual abuse, and 20.3 % reported physical abuse in their last five shifts*.* Psychiatric nurses report among the highest violence victimization rates of all types of nurses [3, 5, 9].

The short or long-term exposure to any type of violence can result in negative outcomes for nurses and organizations. For nurses, there may be both physical and psychological consequences. The psychological outcomes may include anger, fear or anxiety, post-traumatic stress disorder (PTSD) symptoms, guilt, self-blame and shame [2], decreased job satisfaction and increased intent to leave the organization [10], lowered health related quality of life (HRQoL) [11], and in some cases, physical outcomes include injuries, and temporary or permanent disability [12].

At the organizational level, if workplace violence occurs, negative outcomes may include: high staff turnover and difficulty with nurse retention [13, 14], decreased morale, hostile work environments [12], nurse absenteeism, more frequent medical errors, more workplace injury claims [15, 16], greater costs due to disability leaves, and reduced quality of patient care [6, 12]. The costs associated with both short and long-term disability leaves and workplace injury claims have been found to be substantial for organizations and account for approximately 30 % of the overall costs of ill-health and accidents [17]. Statistics Canada reported that 33 % of workplace violence incidents occur in health care and social service settings compared to 14 % occurring in accommodation or food services, and 11 % occurring in educational services. In Ontario, it has been reported that the health care sector has the highest incidence of workplace violence with 8 % of lost time injuries caused by violence and aggression [18]. A previous examination of Ontario Worker’s Compensation Board claims of injuries due to workplace violence reported that the number of days lost averaged 2500 per year with costs estimated at $300, 000 per year [19].

Despite the increasing magnitude of the phenomenon and the identification of it as an occupational hazard within hospitals around the world, few Canadian studies have explored this topic using a qualitative approach to describe and understand the experience of patient violence from the psychiatric nurses’ perspectives [20]. It appears that the majority of violence research in this field has focused on the epidemiology of workplace violence, various risk factors and outcomes of the event in multiple settings. This literature inadequately describes the experiences of patient violence through the lens of psychiatric nurses working in inpatient settings who are deemed to be at much greater risk of experiencing violence. Another gap within the literature on violence is the lack of conceptual clarity of what constitutes patient violence [3, 8, 13]. Therefore this study describes psychiatric nurses’ personal experiences of patient violence within the context of their inpatient work to provide a more holistic and rich description of the phenomenon of patient violence which may inform future clinical practice and policy. This information will identify the knowledge and skill needs of nurses working in this context, and identify the potential barriers inherent in current practice at the clinical and organizational levels.

This exploratory study addressed the following overarching questions:

1. How do nurses describe their experiences of patient violence in acute care psychiatric hospital settings?
2. How do nurses describe the professional and personal outcomes of their exposure to and experiences of patient violence?
3. What strategies do nurses describe as influencing current practices of patient violence?

## Methods

An interpretive descriptive (ID) [21] design is an inductive method of qualitative research that involves the formation of a description, but then moves this description beyond the self-evident to further discover potential “associations, relationships and patterns within the phenomenon”[21] (p. 50) with a focus on bringing the analysis back into the context of the practice field. So for example in this study, we focused on identifying potential associations and relationships between a nurse’s exposure to patient violence and the subsequent perceived effects on her/his health, ability to provide care or to engage empathically in the nurse-patient therapeutic alliance. This method also allowed us to uncover patterns of nursing care – or to begin to understand how different contextual conditions might influence how a nurse responds to patient violence. The overarching goal of ID is to address an applied health research question and create understanding that is of practical importance to the applied disciplines, such as nursing [21]. Furthermore, it is a method where findings are created that are relevant for advancing disciplinary knowledge.

Ethics approval was sought and granted by the Hamilton Integrated Research Ethics Board. Informed consent was obtained from all study participants and participants were offered a five dollar coffee card for each completed interview.

### Setting and study participants

This study was conducted within the context of Canada’s universal, publically-funded health care program which provides acute care coverage for all Canadians requiring health services. Registered nurses are trained within Canada in broad-based, unspecialized programs. Nurses were purposively selected to participate in the study if they met the following inclusion criteria: (1) licensed as an RN in his/her province or territory; (2) fluent in English; (3) currently or previously employed within the last ten years as an RN in psychiatric adult inpatient acute care; and (4) experienced any single type (or combination of types) of patient violence. Potential participants who were engaged in any current legal proceedings related to their incidents of patient violence were excluded, as they were legally bound to abstain from discussing their alleged experiences. There were no geographical limitations and eligible nurses from any province or territory in Canada were eligible to participate. Violence in the workplace was defined in this study as any “incident of aggression that is physical, sexual, verbal, emotional or psychological that occurs when nurses are abused, threatened or assaulted in circumstances related to their work” [4] (p. 30). This inclusive definition aligns itself with the Registered Nurses’ Association of Ontario’s definition to provide consistency amongst existing definitions.

The recruitment strategies included: (1) convenience sampling of RNs in one acute care psychiatric inpatient unit located in South Central Ontario; (2) snowball sampling; and (3) study advertisements in the newsletters of relevant provincial and international professional nursing organizations with a focus on mental health. A sample of 12 eligible RNs agreed to participate in the study. However, the specific unit of analysis was each distinct event of patient violence experienced by each participant. The total sample of 12 nurses described 33 events of work-related patient violence encountered during the provision of inpatient psychiatric care.

### Data collection

Data were collected over a nine month period in 2013 using individual, semi-structured interviews facilitated by the primary author (KS) who has clinical expertise in mental health (Table 1). The primary purpose of the interview was to gain an understanding of how nurses define patient violence and to capture their narrative experiences (stories) of violence in the workplace. Secondary data collection involved defining the problem, conducting a needs analysis and completing a comprehensive practice analysis of patient violence. Interviews were conducted individually at a location of the participants’ choosing or by telephone. Telephone interviews have been reported to decrease cost and travel time for participants, increase the ability to reach geographically dispersed participants, and enhance interviewer safety [22]. Chapple [23] also suggests that qualitative telephone data are rich, detailed and of high quality. Participant interviews averaged 60–90 minutes in length.

**Table 1:** Summary of semi-structured interview guide questions

Primary Questions	Examples of probes
“Can you tell me a bit about yourself and what led to your work in psychiatry?”	i. Probe for information regarding their role, responsibilities, work experience, context of the workplace (i.e. how many beds does the unit have, average workload/patient assignment?)
“What do you consider to be patient violence?”	i. Provide definition for patient violence that the study utilizes: When I talk about “patient violence” I am referring any incidents of aggression that is physical, verbal, or emotional that occurs when nurses are abused, threatened or assaulted in circumstances related to their work, but often everyone has their own considerations…
“Can you describe to me your experience (s) with patient violence?”	i. If there are several experiences, ask participant to choose one that “sticks” in their mind the most to start; if there are more than one incident, go through each separately
	ii. What do you think the causes (antecedents) were leading up to the event?
	iii. What was the client’s diagnosis?
	iv. What time of day/night was it?
	v. How many staff were you working with? Is this a typical staffing level?
	vi. What happened during the event?
	vii. How did it end?
	viii. What were your feelings as this occurred and afterward?
	ix. How distressing did you find your experience?
	x. Probe: Compare these to other incidences participant has described
“What impact did your experience have on you personally?”	i. What were the immediate impacts?
	ii. What were the longer term impacts?
	iii. What are the different impacts of verbal vs. physical violence?
	iv. What impact did it have on carrying out your role as an RN after the incident?
	Probe: Compare these to other incidences participant has described
“What practice strategies influence your **current response** (management) to patient violence?”	i. What personal factors influence your response to patient violence?
	ii. Probe for hospital policies, guidelines, unit norms, individual training and strategies
“In your opinion, what unit and organizational strategies facilitate **prevention** of violence?”	i. What do you as an individual do to stay safe and prevent violence? What about the unit as a whole? The organization?
	ii. What are those strategies that increase your risk of violence?
	iii. How confident do you feel in using these strategies?
	iv. Probe: Compare responses and needs to various incidents described by participant
“What do nurses’ need from the unit and/or organization for management of patient violence?”	
“What do nurses’ need from the unit and/or organization following an incident of patient violence?”	i. What support and follow-up did you receive after your patient violence event?
	ii. Was there anything you felt was necessary to support you that did not occur?

### Data analysis

Interviews were audiotaped and transcribed verbatim by the primary author (KS) and subsequently imported into NVivo 10, a qualitative data analysis software program. Data were analysed inductively using a conventional content analysis approach in which the data were gathered and then coded into emergent themes. This thematic data were then constantly revisited after initial coding to ensure that no new themes emerged. During the coding process, a constant comparative approach was used to compare each individual violent event to identify overall attributes and characteristics of the violence experienced by the nurse.

Participants were invited to a second interview to clarify emerging patterns and enhance data saturation (n = 5). Interpretations of the synthesized data also underwent the “thoughtful clinician test,”[21] (p. 84) where findings were reviewed by other researchers and educators with expertise in psychiatry to determine whether the results were plausible and adding rigour to the study by offering a form of data triangulation.

## Results

Our results are organized by first describing the context, then addressing the responses to the three overarching research questions, followed by an overview of five major independent themes that were found within and across all categories of the data, then finally exploring the interpretation of the needs related to patient violence that participants described. Within the subheading “Strategies”, prevention strategies are further described using the framework of primary, secondary and tertiary prevention strategies. Primary prevention strategies are proactive and are aimed at reducing the complex factors that increase one’s risks for violent behaviour. Secondary prevention focuses on the immediate responses to violence and tertiary prevention requires long term approaches that follow the aftermath of violence and which also address the emotional trauma of the victim [24].

### Background context

The median age of the nurse participants was 37.5 years (range 27–57 years; interquartile range = 16.5), with a median of six years of clinical experience (range 4–23 years; interquartile range = 8) in acute inpatient psychiatry settings in three provinces: Ontario (n = 9), Alberta (n = 2) and New Brunswick (n = 1). Eight participants were female, four participants were male. All participants worked in a general hospital on acute inpatient psychiatry units as opposed to within specifically designated psychiatric hospitals. All were licensed as RNs, one had a college diploma, five received baccalaureate degrees in nursing, and six completed post graduate education in nursing. At the time of data collection, seven participants were currently staff nurses, one was an admissions nurse, two were Clinical Nurse Specialists, one was a nurse manager and one was a psychiatric nurse educator. All had worked as staff within a period of five years of data collection. Nine participants were or had been employed full time, two participants were employed on a part-time basis and one participant worked both full and part time.

### Nurses’ descriptions of their experiences of patient violence

#### Types of violence

Within the context of the interviews, the participants described a total of 25 events that were described as physical violence initiated by a patient and directed towards the RN. The types of physical violence involved being chased and cornered, being hit, punched or grabbed, kicked, spit at, strangled, as well as using a weapon or the environment, such as breaking a window, to elicit violence. Four incidents included a combination of these, such as being hit and kicked simultaneously. These experiences were not restricted to only physical violence with many events also involving simultaneous verbal violence (n = 4) in the form of threats, swearing or demeaning comments.

Experiences of verbal violence, encompassing verbal abuse, as well as emotional and psychological violence, were somewhat more difficult to elucidate for most RNs. Many participants described verbal violence generally as there was consensus that being on the receiving end of verbal violence was a very common occurrence. These events ranged from swearing, to threats, intimidation and gestures, to sexually inappropriate comments to mean, spiteful, confrontational or demeaning incidents. The most commonly described experience was that of threats, intimidation or gestures (n = 7 incidents) with the content relating to physical harm or rape.

#### Definitions of violence

Defining patient violence was complex. No one participant had the same personal definition. Participants readily included physical violence in their personal definitions and tended to include incidences that were most threatening or dangerous to their personal well-being over other types of violence that were seen as more benign, such as swearing. For example, many participants initially remarked that verbal violence was such a common occurrence and thus not always considered to be a form of violence, as stated by this participant:

I guess because we work on psychiatry we assume and forget that verbal violence is violence and so we take it serious [sic] to a certain extent, but where other units would do incident reports for something like that, that is our everyday. (RN03)

More complex however was the relationship between a patient's intention and their expression of violent behaviour according to the nurses in this sample. If the act (verbal, physical or other) was perceived as intentional, or purposeful, and not as a symptom of the patient’s illness, nurses defined those actions as a form of patient violence. However if the patient’s actions were viewed as unintentional, or a result of the patients’ illnesses, some nurse participants stated that it could still be classified as violence depending on factors such as: the situation, the prior relationship with the patient, and whether the violent act had a perceived effect on the individual nurse regardless of the type or intent. For the remaining participants, when violence was considered unintentional, it was legitimized as part of their illness and in this way decreased the perceived level of threat and harm; some nurses would define these actions not as violence per se, but instead acts of self-defence on the part of the patient.

### Professional and personal outcomes of nurses’ exposure to and experiences of patient violence

#### Perceived effects of patient violence on nurses

Nurses perceived that exposure to verbal or physical violence had an effect on both their professional and personal activities. Nurses clarified that the emotional and physical sequelae following their exposure to violence affected the nurses’ abilities to carry out their professional nursing roles. A wide range of emotions were expressed by nurses following incidents of physical violence. In the moment of the actual incident, some RNs recalled feeling fearful, in shock or being numb, or alternatively, feeling nothing including sometimes not feeling pain arising from physical assault. One RN, explained:

I don’t know that I was feeling anything. Adrenaline just takes over, you know? I don’t think in that moment I really felt much of anything. I was really beat up and my arm was swollen and black and blue and I didn’t feel that. (RN01)

Nurses automatically adopted “the mode of do what you need to do” (RN01) as the situation unfolded in order to manage the violence. In the moment, when other staff or patients were involved, some participants described focusing their emotions and energy on protecting others. Shortly after the event, nurses reported that they were often able to reflect on their feelings and the immediate emotional response they had to the incident. It was at this point that participants expressed fear, as exemplified by such statements as, “we were terrified” (RN07), “it scared me” (RN01), or “that guy scared the living bejeezes [sic] out of me” (RN06). Not only were participants afraid for their immediate safety, but they feared what could have happened if the situation had ended differently as described below:

What happens if I can’t go home? I don’t go home one day because something’s happened on the unit or what happens if I can’t any longer do my job because I’ve been physically injured? Or I can’t take care of my son? What happens if, you know, I am permanently disabled? (RN07)

Fear was most intensely described when nurses felt there was a chance they could not manage the situation, or in situations that were perceived as being highly dangerous, and when the physical violence was directed primarily at them. It was less intensely felt when violence was directed at colleagues or unit staff as a whole, or when the nurse participant was stepping in to assist and protect other colleagues. The fear of what could have happened if the situation had ended differently was prevalent regardless of whether nurses involved in the situation were physically injured or not.

Anger towards patients was commonly expressed following physical incidents of patient violence. This was especially prevalent when RNs perceived patients as being able to control their behaviour as demonstrated by this participant:

I was *angry*. This patient in particular…because I had already re-directed her numerous times during her stay…and we had already done so many instructions on transfers and safety and falls risk and because of her cognitive impairment she was not able to comprehend these things and she kept acting independently or unsafely and I couldn’t understand why she wasn’t listening so then when she hurt me I was *very* angry at her and it was *her* I was mad at. I was mad at her. I wasn’t accepting this as an illness…it took me a long time to get through that because she looked physically ill so I didn’t equate mental illness with her physical illness. (RN05)

Anger was often also directed at colleagues, such as other nurses and physicians, when nurse participants perceived their co-workers were not engaging in team work and thus contributed to the experience of patient violence.

Immediately after physical violence occurred, and lasting for several days to months, all participants described feeling a “heightened sense of awareness” (RN05) and hyper vigilance. These feelings were not exclusive to occurring only in the workplace, but occurred outside of work as well. After several incidents of patient violence, heightened awareness and hyper vigilance became part of everyday life as this participant explained, “I don’t even think you are aware you’re doing it anymore – scanning a room or you know, where people are, or you’re listening, that kind of thing…It almost becomes second nature” (RN01).

As a result of exposure to physical violence, some RNs described experiencing negative physical health outcomes or injuries. Injuries described included bites, bruises, lacerations, hair loss, and musculoskeletal shoulder and knee injuries. Other physical outcomes related to their exposure to patient violence included being spit at, having water thrown at RNs, headaches, muscle tension, difficulty sleeping and nightmares. Some participants also described poor food and substance use choices, including alcohol and smoking, as a result of experiencing patient violence.

Whether the experience affected the ability to carry out one’s role as a nurse following physical violence seemed to depend on the perceived seriousness of the incident. Some RNs described difficulty concentrating on nursing tasks and unclear thinking after incidents, also being less trusting of the aggressor and keeping “a little more distance” from them (RN11). This in turn influenced a nurse’s ability to respond to subsequent cues of patient distress or agitation and therefore affected their abilities to proactively prevent violence. Participants also expressed feeling less empathy for their patients as this RN explained, “It makes you not be as skillful or compassionate with other patients” (RN04). Many nurses expressed a reduced level of confidence in preventing and managing future episodes of patient violence following these violent episodes. However for a small number of participants, patient violence provided them with a beneficial experience and enhanced their confidence in their abilities to intervene to prevent or manage patient violence as this participant describes,

I’m more confident in what I do now. I’m more confident in addressing things whereas before I might have avoided situations previously…and less naïve about what can really happen. (RN03)

Similarly with physical violence, verbal violence predominantly evoked feelings of fear and anxiety. Nurses explained that this was a result of feeling vulnerable, ill-prepared to effectively manage the abuse, and afraid of how the violence may escalate. RNs described feeling angry and hurt when verbal violence was perceived as a personal attack about themselves as a person, or a personal affront to their body or their role/competence as a nurse. Again, similar to physical violence, participants described feeling a heightened sense of awareness and anxiety following verbal violence. One nurse emphasized that exposure to verbal abuse “increases your anxiety level around that particular patient [who committed the violence].” (RN02)

Several of the female participants described feeling “belittled” (RN04) when verbal violence involved sexually inappropriate comments regarding their bodies or attire. The most commonly perceived outcome related to a nurse’s exposure to verbal violence was his/her ability to maintain the nurse-patient relationship with the aggressor. As the perceived severity of the verbal violence increased, so did the RN’s desire to withdraw or avoid working with the patient often resulting in a request for a change of patient assignment.

Immediately following an experience of either verbal or physical patient violence, all or most RNs sought support from both formal and informal systems. Management was most often identified as providing formal support as follow-up to the incident however participants more consistently identified seeking out informal support from colleagues, family, and friends regardless of the type of violence.

Although required to be completed soon after the incident, hospital safety incident reports were not consistently reported nor completed by nurses. Participants most often identified completing reports for physical violence resulting in actual injury, but often disregarded reports of physical violence that did not result in injury. Similarly, nurses did not formally report episodes of verbal violence despite an obligation to do so. It is likely that some forms of violence may have become so normalized that RNs may not recognize their exposure to violence or be aware of the negative health consequences associated with this exposure.

After the incident occurred, many nurses in the study still needed to provide some level of care to their patients despite being the object of their aggression. This occurred most often when participants remained at work following the incident or during the next few shifts after the aggressive incident. In order to ensure their safety, RNs tended to distance themselves from the patient physically when they remained assigned to this patient as their most responsible nurse. Other RNs declined to work with the aggressors for a few shifts to provide distance, slowly regain some rapport with the patient and regain some of their own self confidence. As one participant who was forced to continue to work with the patient, she states:

I feel like I kind of went more automatic. I did what I felt was ethically and legally appropriate for care, but I can tell you for sure I did *nothing* that was extra for this person. I did not want to spend time with this person. I feared for my own safety and I was upset about what had happened…(RN08)

Often these interactions became less person-centred and more task-oriented perhaps as a self-preservation strategy for the nurses as they had to continue to carry out their role.

Whether the participant had taken extra time off after the incident, was returning from regularly scheduled time off, or was just returning the next day, RNs experienced similar feelings and concerns. They feared how their colleagues would perceive the situation, they worried about whether the patient was still on the unit, and they continually questioned their safety. These feelings of vulnerability continued for several days following these incidents.

Several RNs in the study chose to use the legal system and either pressed charges or were granted restraining orders against the violent patient at the time of the incident, regardless of whether the nurse experienced verbal or physical violence. This often occurred when there were major injuries or emotional trauma to the RN and when the nurse believed that the patient knew right from wrong and had the abilities to control their aggressive behaviours. Some participants described being encouraged by police or their colleagues to press charges against their patients, but others were equally discouraged by their supervisors, or the amount of work, and time that was required.

As a result of incidents with patient violence, nurses described a change in their behaviour, such as being more likely to resort to using medications, and other coercive measures as a way to contain violence, or being less likely to use hands-on approaches to avoid violence. Still others reported being more cautious around patients, trying to think logically and objectively, as well as depersonalizing situations involving verbal violence.

Five participants saw their patients shortly after the violent episode and feared for their safety when they met outside the hospital unit. One RN described that her patient threatened to rape and subject her to other physical forms of violence. A week after the patient was discharged, the nurse saw him while out walking with her husband and explained:

I was afraid for my safety a week later when my husband and I were walking down the street together and there’s that guy. *Seriously!* There’s that guy! I went, “oh my God” and I had sunglasses on and I thought “what do I *do*?”…My husband was talking about something…I just tuned him out. We’re just holding hands and walking along and I’m like “okay, just keep walking,” right? So what I did was walk right past that guy, but I tell you what, in that moment… I was *really* scared. What do you say to your husband? “Oh this guy is a scary guy…” I can’t tell my husband *anything*! I can’t! What am I supposed to say? (RN08)

Management was most often identified as providing formal support in the workplace following a more serious violent event, such as when a staff member was injured, charges were being laid or the participant needed to leave work. There were some RNs that described having a good relationship with their managers or supervisors, and thus had found them to be very supportive following an incident, but more importantly nurses in the study felt most supported when the manager acknowledged and did not minimize the event as explained by this RN, “just having the event recognized as something that was critical and you know, it was traumatic and …they weren’t minimizing it and actually embracing it as something that was not acceptable” (RN06). On the other hand, many other nurses described feeling very angry, unsupported and blamed by their managers. Some RNs never heard from their managers following events of patient violence, while others described receiving a phone call or a brief conversation, which was felt to be thoughtful, but not sufficiently supportive. Others felt blamed by their managers when questioned about the events that occurred:

I know in a reflective sense…[we] are the best tools for preventing situations, but that form of questioning comes to me as so blame-driven. “*You* just were assaulted, *you* just were hurt”, now suddenly you’re asking “what could have *you* done differently”. Not “what could the system have done differently”, it’s what could *you* have done differently…and its always the first question that always comes up…(RN12)

RNs’ long term responses to patient violence were not easily nor frequently elicited by participants. Those that were identified involved legal decisions, emotional components and job decisions. Present study participants described feeling more cynical, jaded and some RNs even remarked upon psychiatric nurses’ use of “dark humour.”

### Strategies nurses described as influencing current practices of patient violence

#### Contributing factors to patient violence

Nurses in this study identified several patient, nursing and environmental factors that were perceived to be contributors to patient violence. The patient-related factors identified were: (1) type of psychiatric diagnosis; (2) patient’s previous violent behaviour, and (3) substance misuse. Nursing-related factors were: (1) communication amongst nursing staff; (2) engagement in debriefing following the violent episode, and (3) the quality of patient assessment. Finally, unit-related attributes that were also deemed to play a role in patient violence were: (1) the availability of nursing staff; (2) limited or restricted physical space; and (3) the availability of activities for patients.

#### Antecedents

Antecedents, or precursors, to violence are situations or conditions that nurses perceive as triggering patient violence. Most of the 33 incidents of violence toward nurses immediately followed interpersonal interactions between nurses and patients, with other incidents reflecting patient-related intrapersonal precursors such as the experience of symptoms. Within the interpersonal interactions it was further categorized into nurse-patient or patient-patient interactions.

#### Strategies

Nurses described the prevention of violence as representing a continuum organized by primary, secondary and tertiary prevention strategies as previously described. Participants identified similar individual nursing practice strategies when dealing with physical and verbal violence irrespective of the RN’s individual experience, background or workplace. Strategies used were dependent upon the level of perceived threat and/or existing escalation of the patient.

As prevention measures moved along the continuum of primary to secondary strategies, the focus shifted from individual nurse strategies to the adoption of a team approach to manage or prevent patient violence. This also necessitated a change from patient-centred strategies intended to keep the patient’s best interest in mind to prioritizing nurse-centred strategies directed toward controlling the situation, and maintaining one’s self-preservation back to patient-centred strategies after the event occurred to prevent any further incidents. Despite the shift to more nursing-focused strategies, RNs believed they were acting within the patients’ best interests.

While many strategies were discussed and implemented by nurses, many participants described these strategies as ineffective at the time or were not implemented in a timely manner prior to an event happening. Interestingly, the majority of RNs had difficulty in identifying any strategies that they perceived would have been successful to prevent the violence in their workplaces.

### Major themes

#### Power and control

The concept of power and control was woven throughout the nurses’ narratives. In current practice, participants saw themselves as being responsible for controlling patients and the environment. Several participants identified that engaging in power struggles and the loss of power and control for the patients were often contributing factors to violence and knowing this, strategized to prevent violence by giving some control back to the patients. This was demonstrated when participants offered choices, and implemented recovery-focused nursing models, such as the Tidal Model, that promoted putting patients in the “driver’s seat” (RN04), but many RNs had difficulty relinquishing this sense of power and control often driven by their own fears of ensuing violence.

The problem with current practice lies in whether or not the nurses thought the patient was in control as it affected how they responded to the violence, as well as the perceived effect of this exposure on their mental health outcomes. When patients were seen as being in control, RNs felt the violence was intentional, and they became angrier, and often blamed the patient for the incident. Participants often felt a loss of control, resulting in helplessness and disempowerment when they perceived being unable to prevent and manage patient violence which in turn affected many participants’ abilities to care for and empower their patients.

#### Stigma

Stigma was evident throughout the narratives of patient violence for all participants. Stigma was seen when nurses held the view that when working in psychiatry, violence should be expected and some nurses struggled with this concept. While they expressed that violence could not be discounted within this population, they also expressed that it is unfair when individuals hold the belief that people with mental illness will be assumed to be more violent.

Stigma also influenced how the nurse experienced violence. Many stated that patient violence exhibited by someone with a diagnosis of a personality disorder was more intentional and they saw the patient as having more control over their behaviour. Some nurses used phrases such as “they’re doing it to get a rise out of you” (RN03) or “they should know better” (RN04). Personality disorders and substance misuse were seen only as controllable “behaviours” (RN02) and not as truly “serious and persistent mental illnesses” (RN05). Nurses tended to be much angrier, frustrated, less tolerant and more affected by patient violence when it involved a patient with a personality disorder or substance misuse.

#### “Part of the Job”

Participants in the study perceived the culture of nursing as one where patient violence should be accepted as part of the job and many felt this belief was unavoidable. This was especially true of verbal violence and many RNs in the study identified the routine nature of violence as not only part of the job, but something no longer worthy of reporting. At the same time they did not want to accept that reality nor accept that violence was tolerable. This had a negative effect on the RNs perceptions of nursing as this participant highlighted:

I *never* thought I would sign up to be…assaulted as a career path. That was something that I never realized that happened so frequently and that it was almost okay for nurses to be beat up all the time or verbally or physically assaulted and I guess that’s normal practice and it’s going to take a lot longer to change that. (RN05)

#### The balance: Nurses’ health and safety versus patient care

Throughout the narratives, nurses were often conflicted between their role of acting as the health care provider who needed to deliver care in the best interest of their patients versus acting in a way that would protect their own health and safety. The RNs in the study often struggled to balance the decision to carry out their role in providing therapeutic care with the idea that “it’s not part of my job to be hurt” (RN05) and engaging in self-preservation activities which caused internal stress. In physical situations driven primarily by fear and low confidence in their ability to prevent and manage patient violence, participants most often chose to preserve their own health and safety at the expense of patient care. For verbal violence, if it was unrelenting, threatening or included personal attacks, RNs again chose to protect their own health and safety. When situations involved patients deemed to be out of control due to illness, often nurses chose to put the patients’ needs first above theirs. Putting the patient first was also more consistently done for the staff who embraced the principles of the recovery model, but all nurses in the study had their own individual cut off points of when the patients’ needs no longer came first.

Ultimately, this balance creates a conflict in one’s role between the duty to care for patients and the duty to one’s self and own safety.

#### Moving forward: Nurses’ perceptions of their needs relating to patient violence

Throughout the current study, RNs described a number of strategies that they routinely utilized in practice to respond to violence with varying success. Changes that nurse participants identified as needing to occur focused on improving the clinical environment, including both the physical environment as well as forming a healthy team atmosphere, and the creation of best practice guidelines for patient violence to alleviate the role conflict related to balancing the duty to self with the duty to care. Additional education was identified as necessary to reduce and mitigate healthcare workers’ stigma towards patients, as well as to improve their knowledge of evidence-based risk assessments, prevention and management interventions as well as the influence of power and control on patient violence. Furthermore, participants identified that organizations need to be open to the exploration of the phenomenon of violence and embrace an open, blame-free environment for nurses to feel comfortable reporting patient violence. They must therefore work with managers and staff at devising strategies to further reduce the incidents of violence while enacting quality patient care. Future research needs to first, and foremost, work towards creating a universal definition of patient violence.

## Discussion

### Nurses’ descriptions of their experiences of patient violence

#### Definitions of violence

The personal definitions that participants described in this study are comparable to previous research by Smith and Hart [25] who similarly reported that seriously ill patients were given a wider scope of acceptability for anger expression because patients were deemed to be unable to control their behaviour and it helped to intellectually explain the patient’s behaviour for the nurses. However, when intent was present and patients were deemed to be in control of their actions, these findings challenge this existing literature and is supported by Jonker, Goossens, Steenhuis and Oud’s [5] who found that nurses perceived patient violence as being unacceptable, and considered destructive or offensive and not serving a protective or communicative function. The current study findings demonstrate the multifaceted complexity and fluidity of participants’ personal definitions of violence relating to a number of factors.

### Nurses’ descriptions of the professional and personal outcomes of their exposure to and experiences of patient violence

#### Perceived effects related to exposure to violence

The perceived effects of exposure to violence as described by the nurses in this study have consistently been demonstrated throughout the existing literature as evidenced by a thorough systematic review conducted by Needham, Abderhalden, Halfens, Fischer, and Dassen [26]. It is possible the long term effects experienced by nurses are long lasting symptoms of PTSD, negative coping strategies or perhaps play a role in chronic work stress and subsequently burnout. Burnout is characterised by three general categories: emotional exhaustion, depersonalization and personal accomplishment [27]. One study of emergency department nurses credited their built up, pervasive anger towards patients as causing them to withdraw and become callous towards patients [28]. The development of these negative, cynical attitudes and feelings about patients is a cardinal factor in burnout, known as depersonalization [27]. Melchior, Bours, Schmitz and Wittich [29] described nurses working with certain patient groups, such as those that are aggressive or suicidal, are at increased risk of burnout. A key aspect in terms of the development of burnout is the amount and degree of contact with patients and if patients give negative feedback (i.e. aggression). It was hypothesized that nurses who continuously focus on the negative aspects of patients developed a more cynical view of human nature.

### Major themes

#### Power and control

Knowing that the loss of power and control for patients may contribute to violence, nurses strategized to prevent violence by giving control back to the patients which is not unlike Henderson’s [30] findings where nurses viewed involving patients in their care as requiring them to give patients information and to share decision-making powers with them; however with the exception of a few participants, the majority of nurses were unwilling to share their decision-making powers with their patients thereby creating a power imbalance between the nurse and patient with little patient input. Henderson identified factors including nurses’ beliefs that they know best, the view that patients lack medical knowledge and the perceived need for nurses to hold on to their power and maintain control. While participants in the current study did not explicitly identify that patients lacked medical knowledge, they did perceive that their actions to take control of situations were acting within the best interest of patients and thus removing their ability to be a part of their own care while maintaining the nurses’ power and control. Another complexity to this issue is that of the overriding demands of the Canadian Mental Health Act (MHA) [31]. The MHA has substantial influence over nursing care which in turn affects partnerships and patients’ perceptions of nurses’ power [31] thus adding to the difficulty of nurses to relinquish their power when the MHA has little or no bearing. It also has the potential to be discouraging for patients to engage with nurses when they are being offered choices and input into their care at one moment to later be relinquished of all control by nurses who are attempting to gain control over an escalating situation [31]. This was evident when nurses in the current study moved through the prevention continuum from primarily patient-centred strategies to more nurse-focussed strategies aimed at taking control when they perceived patients to be losing control. The complexity of balancing safety and patients’ interests was evident. Participants felt a loss of control during violent situations, resulting in helplessness and disempowerment when they perceived being unable to prevent and manage patient violence which is supported by findings from Lanza and Carifo [32] and can be likened to the behaviour of people experiencing intimate partner violence. There is also evidence that disempowered and oppressed groups, such as nurses, attempt to rise from their current status by gaining control over others [33] and thus a possible explanation could be that when nurses feel disempowered and have limited self-efficacy, the desire to exert more power and control over patients is stronger and thus have more difficulty relinquishing this power to gain partnerships with their patients during violent situations.

#### Stigma

It is difficult to find a solution to patient violence until one can understand the stigma that healthcare workers have towards their patients. Healthcare workers, including nurses, are the people that have the most knowledge of psychiatry, and yet are still displaying prejudice and stigma.

Stigma was evident in the current study when nurses held the view that when working in psychiatry violence should be expected implying that patients suffering from mental health issues are more likely to be violent. While this perception may exist for many nurses, this is a much contested issue in the existing literature and there has been no firm evidence that patients experiencing mental illness are any more or less likely to engage in violence [34–36]. Participants also described stigma towards patients suffering from personality disorders, often associating violence with intentional behaviours and not as a sign of their illness, which may be due to the difficult nature of managing the illness as there are limited evidence-based treatments, therapies or resources available [37].

#### “Part of the Job”

RNs perceived the culture of nursing as one where patient violence is accepted as part of the job and many felt this belief was unavoidable. At the same time they were conflicted as the RNs did not want to accept that reality nor accept that violence was tolerable. A number of other researchers have explored this theme and this is found to be a fairly consistent experience of nurses working in psychiatry. Moylan and Cullinan [7] reported nurses in their study indicated multiple reasons why injuries were not officially documented. One of these reasons was that violence in psychiatry was just an expectation and considered a routine occurrence thus not warranting reporting. Whereas Atkinson [38] described that management in healthcare treats violence against nurses as an expected job risk. Lanza, Zeiss, and Rierdan [39] explored the desensitization of nurses to violence and their acceptance of it as part of the job. Repeated exposure to violence may in fact increase the likelihood of desensitization [40]. An interesting finding by Jonker, Goossens, Steenhuis and Oud [5] was that despite the research that mental health nurses are confronted with aggression and violence on a regular basis, the majority of nurses in their study reported their perceptions that they were rarely, or only sometimes confronted with aggression, yet the mean perceived number of incidents was 181 times a year. They questioned whether patient aggression had become such a part of nurses’ daily practices that it was no longer perceived as a major problem anymore indicating that perhaps nurses and management in healthcare are accepting violence as tolerable. This perception is not exclusive to nurses working in psychiatry, but has been found to be a common perception amongst nurses working in other clinical areas and across several countries [15, 41, 42]. However an important difference was the statements by nurses in this study that they do not want to accept violence as tolerable or part of their reality which may indicate a shift towards the realization that violence in the workplace is not acceptable and nurses are becoming more empowered to take a stand.

#### Role conflict

While this particular form of role conflict relating to violence has not been well explored, role conflict in nursing more generally has. Role conflict, a form of job stressor, refers to the perception of incompatible demands being placed on the organizational member [43]. Role conflict can be a major cause of stress for nurses and has been linked as a risk factor to burnout, particularly relating to personal accomplishments [44], and job stress [29, 45]. Unsurprisingly, role conflict has also been linked to a variety of organizationally and personally dysfunctional outcomes such as job dissatisfaction, turnover, lowered productivity, job-related tension, and anxiety [46]. It is possible that role conflict leads to greater job stress, and greater job stress then leads to feelings of burnout and depersonalization and therefore acts as an antecedent to patient violence thus continuing a positive feedback cycle.

Lanza [47] described a similar role conflict in her research relating to nursing and patient violence. Her findings indicated that nurses who experienced patient violence were inclined to want to attend to their own needs and not care for the aggressor. Similarly, Smith and Hart [25] reported that when threats were perceived as high, nurses often disconnected from their aggressive patient by means of withdrawal, transferring blame, shielding, or seeking peer support which was evident amongst the nurses in this study. Lanza [47] also described that nurses are often hesitant about sharing their personal feelings because it conflicts with their professional goals and that nurses were reluctant to press charges or acknowledge feelings of revenge despite this conflict which was also evident in the current study.

### Study contributions

The findings from the study reported here provide a rich, qualitative account of the current experiences of patient violence from within the Canadian context, and describes how patient violence is experienced by nurses and the strategies they employ to prevent and manage violence. It highlights areas of future needs and research from the RNs’ perspectives and can be used to guide the development of interventions related to practice, professional development, education and future research.

### Strengths and limitations of the study

There were several difficulties encountered throughout the process of this study, including difficulty with recruitment due to the sensitive and contentious nature of the study topic and thus a sample size of 12. A larger sample size may have provided saturation for other thematic patterns. There is also a question of whether the RNs most seriously affected by violence are captured within the sample as it is possible that they have left the job, organization or field. Volunteer bias may also exist within the sample as those interested in the topic may be more willing to participate [48] as well as recall bias affecting participants’ ability to recall events before data collection. Triangulating the results against organizational policies, and incident reports could have enhanced some elements of the findings, however was not conducted due to feasibility.

While the sample resulted in only 12 participants, multiple events were explored and described which reflected the diversity of the experiences. As well, each of the RNs described a broad range of experiences across extensive nursing careers in a variety of nursing positions within different hospitals and geographic regions. Maximum variation through multiple recruitment strategies was purposefully sought amongst the sample as the goal of the study was not to evaluate patient violence within only one specific hospital, health care system or region. This heterogeneity of experiences is a strength of the research study as throughout the findings, despite the level of variation, consensus still emerged among participants that this is an important issue that detrimentally affects RNs in this environment. It also demonstrated that the causes, contextual factors and perceived effects related to exposure to violence seem relatively stable across these differences. Many other strategies to enhance the rigour of the study were also employed throughout the research process including the use of the Thoughtful Clinician Test [21] throughout data analysis which enhanced study credibility and demonstrated the stability of these findings. Along with the Thoughtful Clinician Test, a variation of member checking was conducted by sharing a written synthesis of the study data with the participants and inviting them to comment on the accuracy of findings which was valuable as it gave RNs the ability to further explore and clarify the emerging concepts allowing further saturation of thematic patterns. Field notes and a reflexive journal were kept by the researcher throughout the research process enhancing the confirmability, or neutrality [49], of the study which effectively allowed for reflection on personal biases, pre-existing knowledge and questions arising throughout the data analysis process as well as prevented the error of going native where the researcher assumes they understand the phenomenon on the same level as a participant [21]. The credibility and authority of the researcher is also a strength of the study. As previously mentioned, the Principal Investigator conducted all interviews, and has an expertise in acute care inpatient psychiatric nursing therefore having a familiarity with the setting and phenomenon as well as previous investigative and interviewing experience. As all interviews fulfilled the maximum time limit and several RNs were willing to continue past the allotted time for the interviews, it demonstrates both the interviewer’s skills to have participants share their experiences as well as the comfort of RNs in being able to talk truthfully in a safe environment. Throughout data analysis, coding was completed with the assistance of the research team to obtain consensus surrounding thematic content and coding and thus enhance dependability, credibility and confirmability of the research findings through triangulation [49]. This procedure of multiple coding, alongside peer debriefing, also enabled the opportunity for the discussion of alternative interpretations. An audit trail was recorded throughout the research process which enhances the auditability of the study thus allowing other researchers to better assess the quality of the study and allows other researchers to follow the decisions made in order to repeat the study and obtain similar results [49]. The use of NViVO 10.0 software also aided in the auditability of the study as it enabled the analysis process to be more visible and organized, as well as reduced the risk of losing or misplacing any data and thus ensuring all data were included and analysed [50].

## Conclusions

RNs play a fundamental role within the healthcare system. Often described as caring and compassionate, available to assist those most in need [51], but yet are also the target of both verbal and physical violence by patients. While a common experience, exact statistics are rarely considered accurate due to the prevalence of underreporting by nurses. Patient violence goes beyond purely physical injuries and has many negative perceived effects for nurses, patients and organizations. Despite the violent experiences that these RNs recounted throughout the study, many RNs explained that while nursing was not what they expected, they never questioned being an RN in psychiatry. They valued the moments where they felt they could make a difference for someone in a time of need. By the same token, understanding RNs’ experiences and recognizing the need to support RNs in their times of need must be a priority for organizations, as well as researchers. If RNs are not supported in their roles which can affect their ability to maintain their own mental health, how can we expect them to successfully support the mental health of others?

## Abbreviations

RN: Registered Nurse
PTSD: Post-traumatic stress disorder
HRQoL: Health-related Quality of Life
ID: Interpretive description
MHA: Mental Health Act
